# Progress in Solvent-Based Recycling of Polymers from Multilayer Packaging

**DOI:** 10.3390/polym16121670

**Published:** 2024-06-12

**Authors:** Tianmiao Li, George Theodosopoulos, Chris Lovell, Adamantini Loukodimou, Kranthi Kumar Maniam, Shiladitya Paul

**Affiliations:** 1 Materials Innovation Centre, School of Engineering, University of Leicester, Leicester LE1 7RH, UK; tl245@leicester.ac.uk (T.L.); al555@leicester.ac.uk (A.L.); 2 Materials Performance and Integrity Technology Group, TWI Ltd., Cambridge CB21 6AL, UK; george.theodosopoulos@twi.co.uk (G.T.); k.maniam@twi.co.uk (K.K.M.); 3 Materials Performance and Integrity Technology Group, TWI Technology and Training Centre-North East, Middlesbrough TS2 1DJ, UK; chris.lovell@twi.co.uk

**Keywords:** recycling, polymer, multilayers, flexible packaging

## Abstract

Conversion of chemical feedstocks derived from fossil fuels to virgin polymer, manufacturing of plastics in coal-dependent economies, and increasing consumption of virgin polymers for plastics packaging contribute significantly to environmental issues and the challenges we face. Nowadays, promoting sustainable development has become the consensus of more and more countries. Among them, the recycling of multilayer packaging is a huge challenge. Due to the complexity of its structure and materials, as well as the limitations of existing recycling frameworks, currently, multilayer packaging cannot be commercially recycled thus resulting in a series of circular economy challenges. It is undeniable that multilayer packaging offers many positive effects on products and consumers, so banning the use of such packaging would be unwise and unrealistic. Developing the appropriate processes to recycle multilayer packaging is the most feasible strategy. In recent years, there have been some studies devoted to the recycling process of multilayer packaging. Many of the processes being developed involve the use of solvents. Based on the recycled products, we categorised these recycling processes as solvent-based recycling, including physical dissolution and chemical depolymerisation. In physical dissolution, there are mainly two approaches named delamination and selective dissolution–precipitation. Focusing on these processes, this paper reviews the solvents developed and used in the last 20 years for the recycling of polymers from multilayer packaging waste and gives a summary of their advantages and disadvantages in terms of cost, product quality, ease of processing, and environmental impact. Based on existing research, one could conclude that solvent-based recycling methods have the potential to be commercialised and become part of a standard recycling process for polymer-based multilayer packaging. The combined use of multiple solvent-based recycling processes could be a breakthrough in achieving unified recycling of multilayer packaging with different components.

## 1. Introduction

**Figure 1:**
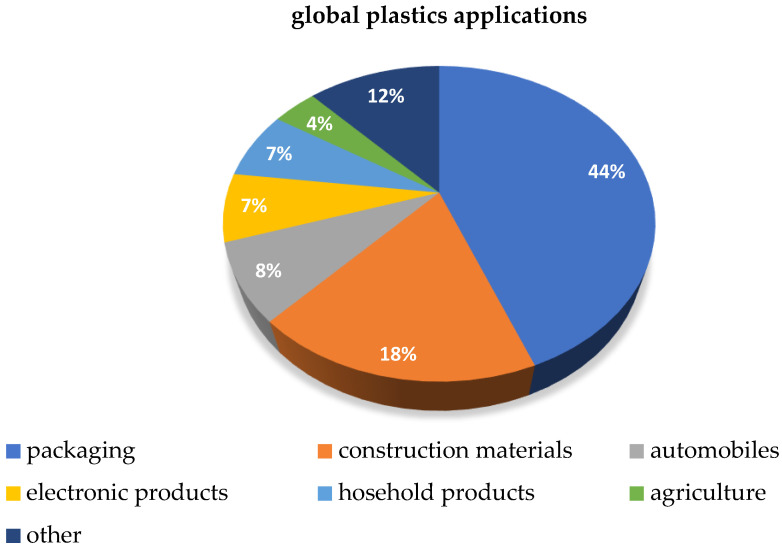
Distribution of the global plastics applications (inspired by [4]).

Since the introduction of the first synthetic polymer, people’s lives have become increasingly dependent on plastic products [1]. Plastics Europe [2] reported that, in 2022, the total global output of plastic products was 400.3 Mt. More than 90% of these plastic products are derived from fossil-based materials [3]. According to a report given in 2022 [4], the total global plastics production in 2021 was 390.7 million tons, the most used field is packaging, accounting for 44% of global demand, followed by construction materials, accounting for 18%, automobiles, electronic products, household products, and agriculture accounting for 8%, 7%, 7%, and 4% respectively, and the remaining 12% covering all other purposes (Figure 1) [4].

Compared to other applications, the characteristics of packaging materials are short service life, large output, diverse components, etc., and the derived post-consumer waste is not easy to collect [5]. Based on the usage, packaging can be divided into two main categories: primary packaging and secondary packaging [6]. Primary packaging is used to protect the product and usually has direct contact with the product. Secondary packaging is used to group and pack primary packages for ease of transport [7]. Normally, mono-material monolayer films are enough to achieve the requirements for secondary packaging applications. Multi-material multilayer structures are mainly used for primary packaging which requires more complex functions than secondary packaging [8]. These functions include containing, sealing, protecting, branding, communicating with customers, etc. [9].

Multilayer packaging can provide adequate protection for sensitive materials and provide convenience for products with short shelf-life. The most widely used field of multilayer packaging is food packaging, which accounts for about 20% of total food packaging in Europe [10]. With the improvements in production technology, the thickness of multilayer packaging has been minimised [11], which resulted in a subsequent reduction in material consumption and production costs [12]. The increasing dependence of consumers on convenience and individually packaged foods has led to a growing trend of the use of multilayer packaging [13]. Subsequently, the continued generation and accumulation of post-consumer waste has become a global challenge [7]. Currently, multilayer packaging cannot be commercially recycled to the level of mono-material packaging (e.g., PET drinks bottles, HDPE milk bottles) since conventional waste management systems are not suitable for sorting and recycling multilayer packaging [14]. This multilayer packaging is considered non-recyclable and only energy recovery or final disposal can be employed for its post-consumer management [15]. It is estimated that polymer-based multilayer packaging accounts for 17–20% of total plastic packaging [15]. According to a report, in Europe, 46% of plastic packaging waste was recycled, 37% was incinerated, and 17% was landfilled [4]. Therefore, it is reasonable to assume that a significant portion (about one-third) of these non-recycled packaging wastes are derived from multilayer packaging waste. This is likely the reason some consumers have negative attitudes towards multilayer packaging [6], and why the industry is also planning to move to mono-material approaches [16]. However, multilayer packaging is usually designed based on the characteristics and requirements of the product being packaged, combining the functionality of different polymers and non-polymeric components [15]. The combined functionality is unlikely to be achieved with any single material, at least not with similar thickness to the designed multilayer packaging. This indicates the irreplaceability of multilayer packaging. In addition, the European Commission aims to make all packaging materials reusable or recyclable by 2030 [17]. Thus, developing appropriate recycling processes becomes the most feasible strategy to managing post-consumer multilayer packaging waste.

This article aims to review the solvents used and researched over the past 20 years for the recycling of polymer-based multilayer packaging, and provide ideas for the commercial recycling of multilayer packaging. The focus of the review is on polymeric materials such as PE, PP, PVC, and PET.

## 2. Recycling Processes

In the most ideal recycling process, the recycled materials have virgin-like properties and purity to the original material and can be reused continuously to produce the original products they were recovered from. This kind of recycling is called “close-loop recycling”. On the contrary, “open-loop recycling” is the process by which the recycled materials can only be used for different applications [8].

There is not a unified classification for the recycling processes. Generally, the recycling processes of plastic products can be divided into mechanical recycling and chemical recycling according to the recycling products. Mechanical recycling does not change the molecular structure of the polymer, while chemical recycling decomposes the polymers into their monomers, oligomers, or other hydrocarbon feedstock [8]. Jefferson Hopewell et al. [18] divided plastic recycling processes into four levels: primary recycling, secondary recycling, tertiary recycling, and quaternary recycling. As shown in Table 1, primary and secondary recycling can be achieved through mechanical recycling processes. The difference between them is that primary recycling products maintain the properties of the original material and can be used as raw materials to produce the same products, while in secondary recycling, also known as down-recycling [8], the products have properties inferior to the original material and can only be used to produce lower-value plastics. Tertiary recycling is chemical recycling, normally using solvents or heat to decompose the polymers found in plastic waste into chemical feedstock for re-production. Quaternary recycling is energy recovery, converting plastics which cannot be recycled in any other methods, into the form of energy through incineration. The recycling world does not consider energy recovery as a recycling method anymore [18].

**Figure 2:**
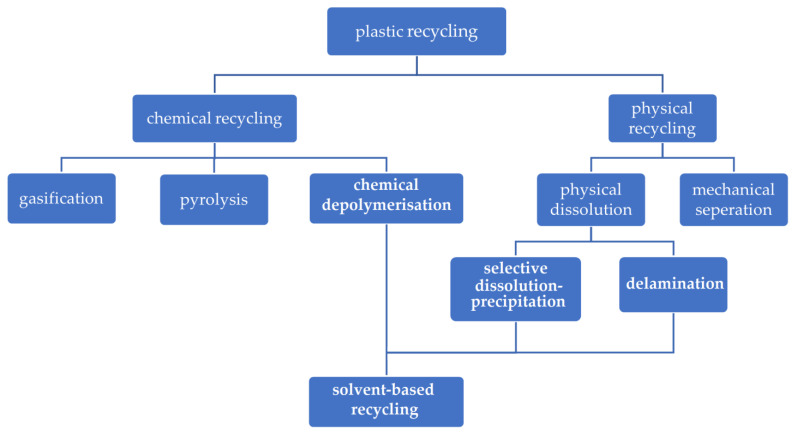
Classification of plastic recycling processes.

In 2021, Collias et al. [19] proposed a new classification for plastic recycling processes containing two major categories: material recycling and chemical recycling. Material recycling includes mechanical recycling and dissolution, and chemical recycling includes depolymerisation, pyrolysis and gasification. Based on Collias’ classification method [19], we clarify the recycling processes into physical recycling and chemical recycling (Figure 2). Physical recycling includes mechanical separation and physical dissolution. Chemical recycling includes chemical depolymerisation, pyrolysis, and gasification. In addition, physical dissolution and chemical depolymerisation are classified as solvent-based recycling. Physical dissolution means to dissolve polymers into a solvent or solvents without changing their molecule structures. Chemical depolymerisation involves breaking polymer chains through chemical reactions between the solvent and the specific bonds in the polymer. In terms of recycling of polymer-based multilayer packaging, there are two main approaches of physical dissolution: delamination and selective dissolution–precipitation (detailed descriptions see Section 3).

This article will review the solvents which have been studied and used in the solvent-based recycling processes to recycle polymers from multilayer packaging. The cost, process difficulty, product quality and environmental impact of these processes will also be summarised at the end of this paper.

## 3. Physical Dissolution

### 3.1. Delamination

**Figure 3:**
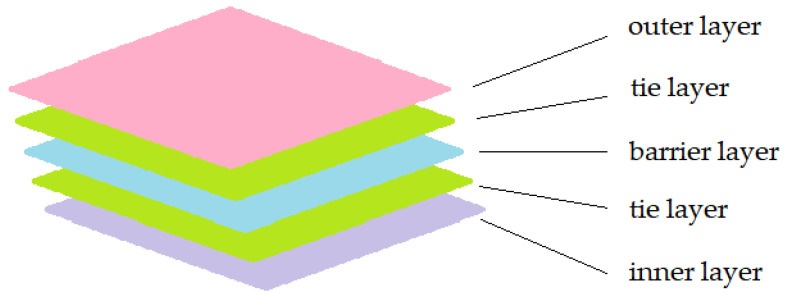
Structure of simple multilayer packaging with outer layer, tie layer, barrier layer and inner layer.

Multilayer packaging often requires the use of adhesives to bond different materials which have different properties. These adhesive layers are called “tie-layers” in a multilayer structure. Commonly used adhesives include acrylics and polyurethanes (PU). Acrylic is more specifically suitable for bonding PE and aluminium, while polyurethane is more versatile and more widely used [20]. A schematic diagram of multilayer packaging is shown in Figure 3.

The delamination processes aim to decompose or dissolve the tie-layers from the multilayer structure separating the target materials and keeping them in their original shape and molecular structure. It can be achieved by removing interlayers or by reactions at the interface [8]. Delamination by dissolving tie-layers is popular because only a small proportion of the structure, (i.e., the adhesive used for bonding dissimilar materials) needs to be removed. The delamination rate mainly depends on ability of the solvent to diffuse in the material and dissolve the tie-layer material [21]. The access surface area is another important parameter affecting the delamination rate. In all experiments reported in the literature, the multilayer packaging samples were cut into strips or pieces (to increase reaction surface area) and thoroughly immersed in selected solvents [20,22,23,24,25,26,27,28,29,30].

Acids (including inorganic acids and organic acids) are one of the most commonly used delamination solvents. In 2023, Šleiniūtė et al. [20] studied the delamination of aluminium-containing multilayer packaging using nitric acid. The role of nitric acid was to dissolve the aluminium layer and break down the structure of PU adhesive. The authors found that the application of ultrasound during the process significantly improved the delamination efficiency of nitric acid, reducing the delamination response duration from 240 min (6 h) to 35 min. They also highlighted that controlling nitrous dioxide gas emissions in the delamination process still requires further research.

Ügduler et al. [22] studied formic acid delamination processes and mechanisms of multilayer packaging containing five constituents. The polymer materials included PET, PE, solvent-based polyurethane (SB-PU) adhesive, and solvent-free polyurethane (SF-PU) adhesive. The authors found that formic acid could diffuse in both polar polymers (e.g., PET) and non-polar polymers (e.g., PE), hypothesising that the good diffusion ability of formic acid may be due to its short alkyl chain. The dissolution of both the SB-PU and the SF-PU in formic acid was proportional to the reaction temperature (50–75 °C) and the formic acid concentration (50–100%). In their further studies, it was found that the solubility of SF-PU in formic acid was smaller than that of SB-PU, also suggesting that it was due to the dense structure of SF-PU, which limits the diffusion of formic acid and its swelling. In addition, the diffusion rate of formic acid in PET-based packages was found to be faster than that in PE-based packages. Thus, the authors concluded that formic acid would be promising for use in large-scale delamination and recycling of PET-based multilayer packaging laminated with SB-PU adhesive.

Apart from organic acids, other organic solvents have also been studied for the delamination of multilayer films. Fávaro et al. [23] reported the use of acetone to delaminate PE-based aluminium-containing multilayer packaging. Delamination was accomplished by stirring in acetone at 50 °C for 4 h. The adhesive used between layers was SB-PU and the delamination products were PE and aluminium-embedded PET. The report demonstrated that PE can be directly recycled by extrusion and PET can be recycled by chemical depolymerisation, which will be reviewed in the next section.

In 2022, O’Rourke et al. [24] studied the delamination mechanism of polyamide (PA)/polyolefin (PO) multilayer packaging by ethylene glycol (EG) oligomers. At a temperature (100–150 °C), diethylene glycol (DEG) could result in glycolysis of the solvent-free polyurethane adhesive in both polypropylene (PP) and polyethylene (PE) based multilayer films, thereby separating the PA and the PO layers. The polymer films recovered by this process contain almost no impurities, making it easy to be reused efficiently.

Solvent mixtures were also employed for the delamination of multilayer packaging in the last decade. In 2014, Zhang et al. [25] studied the interfacial delamination mechanism of an Al-PE multilayer film. The composite film was obtained from a paper-containing multilayer packaging. Before delamination, the paper in the packaging was removed by hydraulic disintegration. The extracted Al-PE multilayer film contained PE, PE adhesive and aluminium foil. A solvent system involving benzene–ethanol–water with a volume ratio of 30:20:50 was used as the separation solution, delaminating PE and aluminium foil. The authors reported swelling of the PE layer and destruction of the PE adhesive layer. Zhang et al. [25] suggested that the delamination mechanism is the interfacial adhesive failure between the PE and the aluminium foil.

Deep eutectic solvents (DES) which are normally non-flammable, non-toxic, and have low vapour pressure [26], have been studied in the delamination processes. In 2020, Nieminen et al. [26] studied the recycling of polyvinyl chloride (PVC) from waste pharmaceutical blister packaging by a DES prepared with choline chloride and lactic acid, where choline chloride acted as a hydrogen bond acceptor and lactic acid acted as a hydrogen bond donor. These two components are considered less hazardous chemicals. The authors found that after recycling, the transparent PVC panels turned slightly white. The explanation for this phenomenon was attributed by the authors to the fact that during recycling the PVC was heated close to its glass transition temperature, which resulted in the increase in the crystallinity of the recycled PVC, thus whitening its appearance. They suggested that re-extrusion can reverse the opacity of the PVC.

Some switchable solvents were also studied as novel solvents to delaminate multilayer packaging in an attempt to ensure the reuse of the solvent without distillation. In 2018, Mumladze and Yousef et al. [27] proposed a method to separate multilayer packaging using a switchable hydrophilicity solvent (SHS), N,N-dimethylcyclohexylamine (DMCHA). After SHS treatment in ultrasound, floating polymer films (Ethylene-vinyl acetate (EVA) and PET) and precipitated aluminium flakes/particles were separated from the solution. The authors suggested that the application of ultrasound treatment accelerated the delamination of the aluminium barrier layer and polymer layers. The ink, paint and sealing layer (PE) dissolved in the SHS solution were extracted by adding water and bubbling CO_2_ for several hours. The properties of DMCHA recovered after removal of CO_2_ were determined to be unchanged from the initial DMCHA. Therefore, the authors believed that the recycling of multilayer packaging by using SHS has potential from both economic and environmental perspectives.

The same research group also studied the DMCHA delamination recycling of PVC and PP from waste pharmaceutical blisters [28]. The recycled polymers were investigated by thermogravimetric analysis (TGA) and differential scanning calorimetry (DSC), and were shown to have good thermal stability and similar glass transition temperatures to the virgin polymers.

Less hazardous amine-free CO_2_-switchable hydrophilicity solvents (ASHS) composed of carboxylic acid and sodium hydroxide (NaOH) were introduced by Cunha et al. [29]. The authors believed that it is possible to use ASHS as a new method for polymer recycling.

In 2023, Vagnoni et al. [30] proposed a method for delaminating PO-Al multilayer film waste using switchable anionic surfactants, which were prepared by mixing carboxylic acid and base (amine or hydroxide) with an equivalent ratio of 1:1.5. The authors concluded that the optimum combination was C12-TEA (lauric acid-triethanolamine), which could efficiently recover PE from de-pulped food and beverage cartons. In addition, they proposed that C12-TEA is better than most of other switchable systems in terms of human health and environmental friendliness.

In order to facilitate packaging delamination, other efforts have also been made in the design of multilayer structures, including the microperforation technique [31], reversible cross-linking adhesives [32,33], water-based adhesives [34], etc.

A summary of the solvents used in the studies reviewed above is shown in Table 2.

The mechanism of delamination is to dissolve or decompose the adhesive layer, which is mainly composed of acrylics or polyurethanes, to separate the main components (e.g., LDPE, PP, aluminium foil, etc.) of the multilayer structural. Since the delamination process is likely to be economically and environmentally friendly, it has become a hot topic of research. A variety of solvents have been studied and tested for the delamination of multilayer packaging:

- Single-component solvents, including inorganic acids (e.g., nitric acid), organic acids (e.g., formic acid), and other organic solvents (e.g., acetone, DEG), etc.
- Mixed solvents, such as benzene–ethanol–water solution system, and choline chloride–lactic acid deep eutectic solvent.
- Recently invented CO_2_-switchable solvent systems; for example, switchable hydrophilicity solvents, and switchable anionic surfactants.

It is worth mentioning that some studies concluded that the use of ultrasound can significantly accelerate the delamination process.

### 3.2. Selective Dissolution–Precipitation

The mechanism of selective dissolution–precipitation (SDP) is to dissolve and separate a series of incompatible polymers one after another by using one solvent at different reaction temperatures, or by using different solvents. The recovery of target polymers is normally achieved by precipitation using anti-solvents, or by solvent evaporation [35]. In real applications of SDP to recycle mixed polymers from a waste stream, all the target polymers should be dissolved and precipitated, aiming to remove impurities and other insoluble plastics [35].

Tetra Pak^®^ is a common multilayer packaging for liquid food [36]. This widely used beverage carton normally consists of 75 wt% of stiff paper, 20 wt% of low-density polyethylene (LDPE) and 5 wt% of aluminium foil [36]. The paper can be recycled through hydropulping, while the LDPE and aluminium foil remain laminated [37]. In order to further separate the LDPE and the aluminium, Georgiopoulou et al. [36] used xylene to dissolve the LDPE from the Al-PE laminates, followed by filtration to recover the aluminium foil, and then precipitated the LDPE using isopropanol as an antisolvent. In their work, the outer PE layer was also dissolved and precipitated. Unlike the fine powder LDPE recovered from Al-PE laminates, the recycled product of the outer PE layer was in the form of lumps. Therefore, the authors believed that the selective dissolution–precipitation process using xylene and isopropanol to recover LDPE is not suitable to remove impurities such as printing inks. They suggested that for high-quality recycling of LDPE, pre-separation of Al-PE laminates and outer PE is necessary.

Samorì et al. [37] tried different sustainable solvents to recover LDPE and aluminium from de-pulped multilayer packaging. The solvents were used to dissolve the LDPE layer and then separate the multilayer structure into single components. The solvents used were biodiesel, 2-methyl tetrahydrofuran (2-MeTHF), and cyclopentyl methyl ether (CPME). The LDPE was recovered by adding ethanol as an anti-solvent in the biodiesel-based process or by distilling the solvent under vacuum for the other two processes. The authors concluded that among the three solvents, CPME performed best for LDPE solubility and recovery, followed by biodiesel and 2-MeTHF. They believed that the results of their work could widen the choice of solvents for recycling plastic waste.

**Figure 4:**
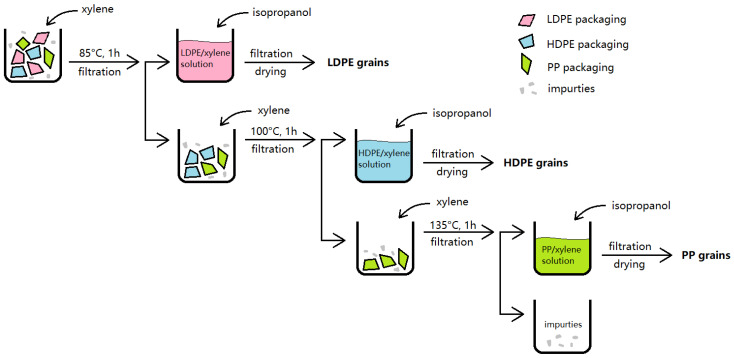
Selective dissolution–precipitation process for the separation of mixed polyolefins waste (inspired by [35]).

A number of publications also reported the recycling of single or mixed polymers using the method of selective dissolution–precipitation [35,38]. Although multilayer packaging was not included in these papers, the solvents and the experimental parameters are valuable for reference. Pappa et al. [35] used xylene and isopropanol as solvent and anti-solvent to dissolve and precipitate mixed LDPE, HDPE and PP. The separation procedure is shown in Figure 4 [35]. LDPE, HDPE and PP were dissolved in turn by xylene at 85 °C, 100 °C and 135 °C. The authors reported that the polymers recovered by the xylene/isopropanol system retained their value, therefore, they believed that this technique could be applied in the recycling of mixed plastic waste.

Achilias et al. [38] studied the dissolution–reprecipitation recycling of a series of commonly used polymers from plastic packaging materials. The polymers included LDPE, HDPE, PP, polystyrene (PS), PET and PVC. A summary of the optimum solvent and dissolution–reprecipitation parameters obtained from their research is shown in Table 3 [38]. D-limonene as an environmental-friendly solvent was used for the recovery of PS. The authors claimed that the advantages of using this solvent could be the low dissolution temperature, high solubility, low dissolution time and high selectivity. In addition, the D-limonene can be removed by vacuum distillation thus preventing the usage of an anti-solvent.

Aiming at the deconstruction of multilayer films, Walker et al. [39] addressed a unique strategy named solvent-targeted recovery and precipitation (STRAP). Walker et al. demonstrated the STRAP process by separating a three-component multilayer film consisting of PE, ethylene vinyl alcohol copolymer (EVOH) and PET. The PE fraction was dissolved by toluene at 110 °C and precipitated by acetone. The EVOH was dissolved by dimethyl sulfoxide (DMSO) from the remaining solid and was precipitated by water. The remaining PET was filtered, washed and separated directly without dissolution and precipitation. The authors reported that the polymers recovered by the STRAP process were in a chemically pure form while being cost-competitive with the corresponding virgin materials [39].

Further research was conducted employing the STRAP process. Sánchez-Rivera et al. [40] studied the separation of multilayer films with more complex components in 2021 proposing temperature-controlled STRAP to reduce the use of anti-solvents in the recycling of multilayer plastic films. They compared two STRAP processes separating a PE, EVOH, PET and EVA (minor) multilayer film. In STRAP-A, PE and EVOH were selectively dissolved by toluene and DMSO and precipitated by anti-solvents of acetone and water, respectively. This process did not separate PE and EVA adhesive layers as they were dissolved and precipitated together which influenced the purity of the recovered PE. In STRAP-B, PE was precipitated by cooling the solution from 110 °C to 35 °C, while the EVA remained in toluene and was later precipitated in acetone. EVOH was dissolved by the mixture of 60% DMSO–40% water (*v*/*v*) and precipitated by cooling the solution from 95 °C to 35 °C. The authors found that the yields of the recovered polymers were similar from the two STRAP processes, while STRAP-B, using less anti-solvent, was even more efficient in the separation of PE and EVA. Therefore, they came to the conclusion that the temperature-controlled polymer dissolution and precipitation is a promising optimisation to make STRAP cost competitive and environmentally friendly [40,41].

In the same paper, Sánchez-Rivera et al. also reported a STRAP-C process for separating a polyethylene terephthalate glycol (PETG), PE, EVOH, PET and EVA multilayer film [40]. A PETG selective separation procedure was conducted before STRAP-B. The PETG was dissolved by a solvent mixture of 60% dimethylformamide (DMF)–40% tetrahydrofuran (THF) (*v*/*v*) and precipitated by n-propanol. In 2023 [41], they published a study in the separation of a multilayer printed film composed of PE, EVOH, PET and PU-based inks. After the removal of PE and EVOH, the remaining PET and PU inks were separated in γ-Valerolactone (GVL) which successfully removed the PU inks.

The solvents which have been studied for the selective dissolution–precipitation are summarised in Table 4.

As for the selective dissolution–precipitation method, the commonly used solvents for recycling polyolefin components (including LDPE, HDPE, PP, etc.) from multilayer materials are toluene and xylene, and the anti-solvent can be isopropanol or n-hexane. Some sustainable solvents, such as biodiesel, 2-MeTHF and CPME, have also been explored for the dissolution and separation of LDPE. Except for biodiesel, where the dissolved LDPE requires the addition of ethanol for precipitation, the other two solvents do not require the use of anti-solvents. In addition, the STRAP recycling processes developed for multilayer packaging films laminated with PE, EVOH, PET and EVA have also received certain attention and research. Due to the complexity of the composition and structure of post-consumer multilayer packaging waste, the recycling processes of involving selective dissolution–precipitation are still in the development and experimental stages.

## 4. Chemical Recycling

Chemical recycling refers to a recycling method that causes changes in the chemical structure of plastics during the recycling process [42]. The products of chemical recycling are normally monomers, oligomers and gaseous reactants [43]. They can be used as feedstock to reproduce the original polymer or as basic chemicals in other applications [44].

From the perspective of recycling products and the ease of their reprocessing, physical recycling is preferred. Because physical recycling maintains the polymer structures, making them can be directly used for reprocessing and reproduction. However, it has certain limitations, such as the extensive requirements in pre-sorting, cleaning and polymer chemical ageing, and physical/mechanical degradation. The most important constraint of physical recycling is that the product obtained is strongly affected by contaminants [45]. The quality of the physically recycled polymers, depending on the applied method, is not likely to be maintained after one or several recycling circles, resulting in a material, which is unable to return to a close-loop production line of its original products [46].

Chemical recycling is classified as tertiary recycling, meaning that the priority of chemical recycling is ranked after primary and secondary recycling which can be achieved by mechanical/physical recycling. Since the reactions involved in chemical recycling often require high temperature and/or high pressure and long reaction times [47,48], chemical recycling is considered energy and cost intensive [49]. An advantage of chemical recycling is that it can be used to recycle and repurpose contaminated and mixed plastic waste, therefore it is regarded as complementary to mechanical/physical recycling [50]. The recycled highly pure monomers can be used for the production of high-quality requirement applications such as food packaging [42], as polymers synthesised by these recycled monomers can retain virgin-like properties.

As mentioned in Section 2, there are three categories of chemical recycling: chemical depolymerisation, pyrolysis, and gasification [46]. Pyrolysis and gasification refer to the decomposition and/or the reformation of polymers by heat. It can be used for both addition polymers including PE, PP, PVC, and condensation polymers. Chemical depolymerisation normally requires the use of solvents to break the molecular structure of the target polymer in plastic waste, and to recover them into monomers or oligomers [51], and is normally applied for condensation polymers [50]. In Section 4.1, the review is only focused on the solvents which have been used and studied for chemical depolymerisation of PET, as it is one of the most common components used in multilayer packaging.

### 4.1. Chemical Depolymerisation of PET

In a report released in 2022 [52], Europe produced 4.14 Mt of PET, 2.4 Mt of the PET was recycled with about 90% from PET bottles. However, the recycling of non-bottle PET waste, is still a problem. These PET waste streams are difficult to recycle due to their wide range of colours and complex structures [53].

The chemical depolymerisation of PET has been extensively studied. The main processes include hydrolysis, alcoholysis, glycolysis, and recently aminolysis and ammonolysis [43]. Processes have been used commercially for PET bottle recycling [43,54]. Recently, chemical depolymerisation of PET in multilayer packaging has also received increased attention. Unlike recycling single-material PET bottles, other components in multilayer packaging also need to be taken into account when determining reaction conditions and selecting solvents.

Ügdüler et al. [53] studied the recycling of multilayer and coloured PET plastic waste through alkaline hydrolysis. The depolymerisation of PET was achieved under mild reaction conditions (80 °C, atmosphere pressure). A co-solvent comprised of 60:40 vol% ethanol–water mixed with 5 wt% NaOH was reported as the optimum combination resulting in 95% conversion of PET within 20 min. The recycled product in the solvent was disodium terephthalate, which was then converted and precipitated by sulphuric acid in the form of solid terephthalic acid (TPA). Thanks to the mild reaction conditions, the other polymers in the multilayer structure were maintained without decomposition and could be easily recycled by filtration. The authors believed that this process has the potential to be scaled up as a chemical recycling process for complex PET plastic waste with some adjustments to meet the environmental sustainability requirements.

**Figure 5:**
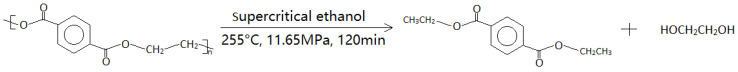
Alcoholysis mechanism and reaction conditions of PTE with supercritical ethanol (inspired by [23]).

In the work reported by Fávaro et al. [23], PET was recycled via ethanolysis of an aluminium-embedded PET film, which was prior delaminated from a PE-based aluminium-containing multilayer packaging by acetone. In this procedure, ethanol was used under supercritical conditions and the products of PET depolymerisation were diethyl terephthalate (DET) and EG. The depolymerisation conditions and products are shown in Figure 5. The recovered diethyl terephthalate can be precipitated by water, and EG can be purified by ethanol and water distillation. The authors believed that this recycling method can be considered environmentally friendly as both the acetone and ethanol used in the process can be recovered by distillation.

Due to the low volatility of diol solvents, glycolysis is considered one of the best methods for PET recycling [55]. The most commonly used diol is EG, which normally depolymerises PET into one monomer, bis (2-hydroxyethyl) terephthalate (BHET), with the assistance of a catalyst. The most efficient and widely used catalyst for PET glycolysis is zinc acetate [56].

Aguado et al. [57] studied the glycolysis of complex PET waste using excess EG in the presence of zinc acetate as a catalyst. The yield of the depolymerisation product BHET was 79–88% depending on the type of PET waste using the reaction conditions, i.e., 195 °C, 150 min. Fourier transform infrared spectroscopy (FTIR) and DSC were employed for material characterisation with results showing the production of high purity BHET. The EG used in the process could be distilled and reused with fresh EG to balance the negative economic and environmental impacts of the excessive use of EG in the process.

Glycolysis of PET, using a large excess of both EG and zinc acetate catalyst, was studied by López-Fonseca et al. [58]. Complex PET waste materials, including highly coloured and multilayered PET, were depolymerised into BHET which was proven by DSC, FTIR and ^1^H NMR to have the same high purity as the BHET obtained from pure PET. The authors claimed that glycolysis could be a suitable process for the chemical recycling of complex PET waste, although further purification may be necessary to produce high quality BHET.

In 2013, Aguado et al. [59] compared alkaline hydrolysis and glycolysis of PET complex waste. The composition of the waste was mainly PET with the presence of other materials including PE, PP, PA, paper, aluminium, etc. In the alkaline hydrolysis process, an aqueous solution of NaOH was used to achieve the depolymerisation of PET into disodium terephthalate and EG. An aqueous solution of HCl was used to precipitate the TPA from the solvent. In the glycolysis process, EG and zinc acetate catalyst were used and the depolymerisation product was BHET. TPA and BHET products were verified by DSC and FTIR as having equivalent properties to the reference PET.

In a study reported by Kulkarni et al. [60], sub- and super-critical water were employed to recover pure aluminium from PET-containing waste composite laminates. The reactions were conducted in a capped stainless-steel tube to maintain pressures under different reaction temperatures. During the reaction, the PET underwent hydrolysis and depolymerised into EG and TPA. The result indicated that 310 °C was the optimum temperature where the EG yield reached maximum. The authors believed that applications of sub-/super-critical water technology has potential in the industry because water is a green solvent.

Based on the studies mentioned in this section, a summary of the solvents used for chemical depolymerisation is shown in Table 5.

Chemical depolymerisation is mainly available for condensation polymers. In this paper, only the solvents used in the chemical depolymerisation of PET were reviewed, as PET is the most commonly used condensation polymer in multilayer packaging.

Similar to the chemical recycling of PET bottles, the depolymerisation of PET in multilayer packaging also follows the mechanisms of hydrolysis, alcoholysis, glycolysis etc. The monomers obtained from these three processes are TPA, DET and BHET, respectively. The commonly used solvent for alkaline hydrolysis is aqueous NaOH solution. In this process, strong acids such as sulphuric acid and hydrochloric acid, are normally required for precipitation of the dissolved disodium terephthalate to solid TPA. The most mentioned alcoholysis method is methanolysis. The primary chemical depolymerisation method for PET in multilayer packaging is ethanololysis conducted under supercritical conditions of ethanol, with EG used as the solvent for PET glycolysis, and normally with the assistance of zinc acetate catalyst.

## 5. Discussion

Based on the works reviewed earlier in this paper, the solvent-based processes for recycling multilayer packaging: delamination, selective dissolution–precipitation, and chemical depolymerisation, are discussed and compared from the perspectives of cost, process difficulty, product quality and environmental impact. A summary is shown in Table 6. It is worth noting that this is only a rough summary and comparison. For each individual recycling process, a detailed life cycle cost (LCC) analysis is required to determine the accurate energy consumption and the environmental impact.

Among the three recycling methods, delamination is the most cost-effective because only the adhesive layer, taking the smallest proportion of the multilayer structure, needs to be dissolved or decomposed. The amount and type of solvent can be minimised. Therefore, it is more likely to have a smaller impact on the environment. Generally, only one solvent is often required. However, the use of multiple solvents is not uncommon. Compared with the other two recycling methods, proper sorting of recycled products after delamination is necessary. Commonly used methods include, sink–float separation, which separates the multilayer components based on their different densities, but certain errors may affect the quality of the recycled products. For printed multilayer packaging, additional deinking steps could increase the complexity of the process. In general, delamination is in the mainstream of research in the field of multilayer packaging recycling.

The selective dissolution–precipitation method is currently receiving increasing attention in the recycling of multilayer packaging as a mechanical recycling method that does not change the chemical structure of the polymer. Due to the complexity of the multilayer packaging structure, the selection of solvents and the design of the process flows are difficult. The use of solvents and anti-solvents and repeated precipitation and filtration greatly increase the cost of the process. In addition, the environmental impact of the solvents used also needs to be considered. Compared to delamination, the target polymers separated by selective dissolution–precipitation could be of higher purity, and some processes can even recycle the polymers in granular phases, which can be directly used in the production of new products, eliminating the cost of re-granulation.

Chemical depolymerisation is the most energy-intensive among the three recycling methods, and accordingly, the cost is relatively high. Chemical reactions involved in the recycling processes often require high temperatures, catalysts, and even high pressures, rendering the process design complex. For example, glycolysis requires the use of excessive amounts of solvent, which can be economically and environmentally challenging to manage. The most important advantage of chemical depolymerisation is high purity of the recycled products and that it could be less affected by the number of recycling times and contamination. As reported by Fávaro et al. [23], the combined use of chemical and other recycling methods may offer more quality and cost options for the recycling of multilayer packaging.

## 6. Summary and Conclusions

With the high demand of multilayer packaging, the disposal of post-consumer multilayer packaging has also received increasing attention. Close-loop recycling, in which the recycled polymers can be transformed back into the original packaging materials, is undoubtedly the ideal disposal method in terms of environmental and sustainability. Products made through down-cycling, to some extent, can also be accepted by consumers as daily necessities and less demanding applications (such as bin bags). Chemical recycling is another effective way to recycle and reproduce high-quality products; however, at higher production cost and requirements. Energy recovery is no longer classified as a recycling approach in the recycling world. Landfilling is a disposal method that should be avoided at all costs; nevertheless, it is still the final destination for the vast majority of post-consumer multilayer packaging.

Due to the diversity of solvent selection, solvent-based recycling can meet the requirements of different recycling aims. This paper reviewed solvents used for polymer-based multilayer packaging in the processes of three solvent-based recycling methods: delamination, selective dissolution–precipitation, and chemical depolymerisation. From this review, we can find that there have been some lab-scale studies on processes for recycling polymers from multilayer packaging. Some of them have achieved good recycling results with high product quality. For the next step of research, there could be three directions to consider: first, continue to explore safer and more efficient solvents for different polymer components in multilayer packaging; second, based on the solvents which have been studied, explore the feasibility of scaling up the processes; third, develop a solvent-based recycling framework specific to or applicable to multilayer packaging, possibly including independent sorting systems.

Different recycling processes have their advantages and disadvantages. Breaking down the barriers between processes, selecting different solvents, and designing processes based on the different components in post-consumer multilayer packaging waste is expected to generate novel solutions for multilayer packaging recycling.

## Figures and Tables

**Table 1:** Common classification, approaches and examples of plastic recycling (inspired by [18]).

Classification	Approach	Examples of Recycled Products	Applications
Primary recycling	Mechanical recycling	Virgin-like PET granules	New bottles
Secondary recycling	Mechanical recycling	Lower value plastic granules	Trash bags; pipelines
Tertiary recycling	Chemical recycling	BHET (monomer of PET)	New PET polymer
Quaternary recycling	Energy recovery *	-	Power energy

* Incineration for energy recovery is no longer classified as recycling in Europe [18].

**Table 2:** Summary of solvents used in delamination processes.

Classification	Solvents	Other Conditions	References
Physical dissolution-delamination	Nitric acid	Ultrasound	[20]
	Formic acid	-	[22]
	Acetone	-	[23]
	DEG	-	[24]
	Benzene–ethanol–water (30:20:50)	-	[25]
	Choline chloride–lactic acid DES	-	[26]
	DMCHA SHS	Ultrasound	[27,28]
	C_12_-TEA	-	[30]

**Table 3:** Optimum solvent/anti-solvent and dissolution temperature for studied polymers (inspired by [38]).

Polymer	Solvent	Antisolvent	Dissolution Temperature
LDPE	Xylene	n-hexane	100 °C
HDPE	Xylene	n-hexane	100 °C
PP	Xylene	n-hexane	140 °C
PS	D-limonene	-	25 °C
PET	benzyl alcohol	methanol	180 °C
PVC	Dichloromethane	methanol	40 °C

**Table 4:** Summary of solvents used in selective dissolution–precipitation processes.

Classification	Target Polymer	Dissolution (Solvent)	Precipitation (Antisolvent or Operation)	References
Physical dissolution–selective dissolution–precipitation	LDPE	Xylene	Isopropanol	[35,36]
		biodiesel	ethanol	[37]
		2-MeTHF	Distilling the solvent	[37]
		cyclopentyl methyl ether	Distilling the solvent	[37]
		* xylene	n-hexane	[38]
	PE	toluene	acetone	[39,40]
	HDPE	Xylene	isopropanol	[35]
		* xylene	n-hexane	[38]
	PP	Xylene	isopropanol	[35]
		* xylene	n-hexane	[38]
	PS	* D-limonene	Distilling the solvent	[38]
	PET	* benzyl alcohol	methanol	[38]
		GVL (remove PU ink)	-	[41]
	PVC	* dichloromethane	methanol	[38]
	EVOH	DMSO	Water	[39,40]
		DMSO (60%)–water (40%)	Cooling from 95 to 35 °C	[40]
	PETG	DMF (60%)–THF (40%)	n-propanol	[40]

* dissolution of commercial waste packaging materials, may not be multilayer structures.

**Table 5:** Summary of solvents used in chemical depolymerisation processes.

Classification	Solvent	Other Chemicals	References
Chemical depolymerisation	5 wt%NaOH in ethanol(60%)-water(40%)	-	[53]
	Supercritical ethanol	-	[23]
	EG	Zinc acetate catalyst	[57,58]
	NaOH aqueous solution	HCl aqueous solution for precipitation of TPA	[59]
	Sub-/super-critical water		[60]

**Table 6:** Summary and comparison of different solvent-based recycling processes.

Solvent-Based Recycling Process	Cost	Process Difficulty	Product Quality	Environmental Impact
Delamination	low	low	low-medium	low-medium
Selective dissolution–precipitation	medium-high	medium-high	low-medium	medium-high
Chemical depolymerisation	medium-high	medium-high	high	low-high

## Data Availability

No new data were created or analysed in this study. Data sharing is not applicable to this article.

## Nomenclature

**Abbreviations**	**Definition**
^1^ H NMR	Proton nuclear magnetic resonance
2-MeTHF	2-methyl tetrahydrofuran
Al	Aluminium
ASHS	Amine-free CO_2_-switchable hydrophilicity solvent
BHET	Bis(2-hydroxyethyl) terephthalate
C_12_-TEA	Lauric acid-triethanolamine
CPME	Cyclopentyl methyl ether
DEG	Diethylene glycol
DES	Deep eutectic solvent
DET	Diethyl terephthalate
DMCHA	N,N-dimethylcyclohexylamine
DMF	Dimethyl formamide
DMSO	Dimethyl sulfoxide
DSC	Differential scanning calorimetry
EG	Ethylene glycol
EVA	Ethylene-vinyl acetate
EVOH	Ethylene vinyl alcohol copolymer
FTIR	Fourier transform infrared spectroscopy
GVL	γ-Valerolactone
HCl	Hydrochloric acid
HDPE	High-density polyethylene
LDPE	Low-density polyethylene
NaOH	Sodium hydroxide
PA	Polyamide
PE	Polyethylene
PET	Polyethylene terephthalate
PETG	Polyethylene terephthalate glycol
PO	Polyolefin
PP	Polypropylene
PS	Polystyrene
PU	Polyurethane
PVC	Polyvinyl chloride
SB-PU	Solvent-based polyurethane
SF-PU	Solvent-free polyurethane
SDP	Selective dissolution–precipitation
SHS	Switchable hydrophilicity solvent
STRAP	Solvent-targeted recovery and precipitation
TGA	Thermogravimetric analysis
THF	Tetrahydrofuran
TPA	Terephthalic acid
